# Peer worker involvement in N. Macedonia: Reflections and lessons learned

**DOI:** 10.1192/j.eurpsy.2021.1883

**Published:** 2021-08-13

**Authors:** M. Milutinović, G. Kalpak, L. Novotni, S. Bajraktarov, A. Novotni

**Affiliations:** Department For Affective Disorders, University Clinic of Psychiatry, Skopje, North Macedonia

**Keywords:** peer worker, N. Macedonia, community mental health, RECOVER-e project

## Abstract

**Introduction:**

For the first time in N. Macedonia we had the experience to include peer workers as an equal members in the newly formed community mental health teams. For the purpose of the RECOVER-e project we engaged 2 patients to be the new peer workers.

**Objectives:**

To evaluate the initial period of peer worker involvement in the community mental health teams and to identify the next steps for them.

**Methods:**

Conducting interviews with the peer workers.

**Results:**

One important aspect that is worth mentioning is their motivation to continue their work in the community mental health teams and continue to improve. Another finding is their will to establish a user led organisation. A user led organisation in the field of mental health in our country is rare to be find, with only a couple of them functioning in N. Macedonia. So if our peer workers can make it, it would be a great step in the right direction.

**Conclusions:**

Overall, taking into consideration all that the peer workers have achieved in the last period, including the ups and downs of being the first official peer workers in N. Macedonia, they are looking forward to improve their knowledge and continue building their practical experience. A great plus of it all is that they have a financial stability for this troubled pandemic period.

**Disclosure:**

No significant relationships.

